# Smart prevention device for foot infection

**DOI:** 10.1186/1753-6561-5-S6-O29

**Published:** 2011-06-29

**Authors:** M Rocklinger, P Vacherand, F Brönnimann, A Mathieu, A Stéphane, Z Pataky

**Affiliations:** 1 POWERSENS, Plan-les-Ouates, Switzerland; 2 Geneva University Hospital HUG, Geneva, Switzerland

## Introduction / objectives

Each year 30 millions of foot ulcers occur worldwide and 56% of them will become infected. These infected foot ulcer represents the leading cause of lower-limb amputation in the world and shortens dramatically the lifetime of people.

The reduction of high plantar pressure in patients with peripheral neuropathy such as diabetic people is mandatory for the prevention of foot ulcers and amputations.

The device "Power Insoles" is an electronic shoe insole that monitor plantar pressure throughout the day, and prevent apparition of foot ulcers by informing the patient through biofeedback signals on a smartphone.

## Methods

After a screening of peripheral vascular disease and loss of protective sensation of the foot. Power insoles are prescribed to the patient. These user friendly stand alone insoles measures plantar pressure continuously in real time.

When repeated high pressure points over time occurs, patient is warned with a bio-feedback signal transmitted wirelessly from the insole. The patient is informed visually, by sound and by vibrations through a smart-phone. According to these signals the diabetic can modify his walking gait pattern, decide to rest, or check his feet and/or consult a foot specialist.

The information given by the insoles are also very valuable to medical staff, for diagnostics, choice of treatment, and patient education.

## Results

Recent studies at Geneva University Hospital as shown that subjects suffering from foot neuropathy can modify their foot pressure distribution by offloading at-risk area in response to the biofeedback signal. They were able to find by themself a new walking strategy !

## Conclusion

Thereby, "Power Insoles" are design to reduce the number of foot ulcer infections and improve lifetime and health conditions of many people.

## Disclosure of interest

None declared.

